# Supplementary material for: Ultrasound as a reliable guide for lumbar intrathecal injection in rats: A pilot study
Source: PLoS One. 2024 Sep 5;19(9):e0301567. doi: 10.1371/journal.pone.0301567 (PMC11376581; doi:10.1371/journal.pone.0301567)
Supplement: Attachment — Submitted filename: Response to reviewers.docx [file pone.0301567.s004.docx]

May 30^th^, 2024

Editorial Department of PLOS One

Dear Chief Editor,

Thank you for your response and input for our manuscript entitled “**Ultrasound as a reliable guide for lumbar intrathecal injection in rats: A pilot study**” and considered for publishing by PLOS One.

In accordance with the Data Availability policy, we confirm that all raw data required to replicate the study results are available within the manuscript, attached figures and supplementary video file.

All authors and their affiliations are included in the title page within the main document, with corresponding author indicated.

Furthermore, we would like to address some of the queries from reviewers

**Reviewer #1**

Recommendation 1: The origin of the needle, its length, and its visibility on ultrasound should be stated.

*We have added additional information regarding the origin and length of the needle in the manuscript, specifically line 97–98, needle visibility on ultrasound was described at line 207–210. Thank you for the recommendation*

Recommendation 2: Specifying the level where the spinal cord ends in the Discussion-Anatomy section provides more descriptive information for a safe intrathecal injection site.

*We have added the information regarding where the spinal cord ends in the anatomy section, specifically line 182–190, thank you for your input.*

Recommendation 3:

Technique 1: Wouldn't it be more appropriate to express it as "paramedian sagittal" instead of "longitudinal-sagittal"?

*We agree with the term technically, however given the large probe compared to the tiny vertebra of the rat, we weren’t sure of the exact plane shown was true perpendicular median-sagittal or slightly paramedian-sagittal oblique. We agree to change the terminology to a more general term in describing the probe position, sagittal view.*

Comment 1: While 3 out of 3 successes were reported in technique 2, 1 out of 3 success was reported in technique 1. The use of the paramedian oblique sagittal (PMOS) plane could increase the chance of success by providing visibility of the posterior complex including the ligamentum flavum and posterior dura.

*We agree that PMOS plane was shown to augment posterior complex in human, however it is difficult to determine in rats. Slight adjustment with a large probe compared to the tiny vertebra, it is mathematically difficult to determine whether the plane shown was true median/paramedian. For note, the distance between midline to the edge of lamina was approximately less than 0.5 mm in rats. It required dynamic scanning to obtained the documented view, with recommendation of firstly place the probe at the midline, then identify the lamina and interlaminar space. (Line 99­–102). The probe would be in sagittal plane between the median to paramedian line, facing the interspinous space. Therefore we also recommend this to be performed by an operator which already familiar in using USG in various procedure involving needle puncture which we added in line 285–7.*

Comment 2: The importance of such studies will increase as preliminary examination and/or real time ultrasound will be used to reduce technical difficulties in spinal anesthesia applications with anatomical landmarks in humans, and ultrasound image recording will be evidence in possible legal/judicial procedures.

*The direction of this study were not as a support for preliminary examination and/or real time ultrasound for reducing spinal anesthesia anatomical landmarks and technical difficulties in humans, since the ultrasound uses and advantages in human, especially during neuraxial procedure, were quite established. We rather had the inspiration from that experience for similar application in rats since ultrasound aid had not been demonstrated before and was more challenging to perform in rats. We hoped that this method could give a more feasible, reliable, and objective guide compared to the previous traditional tail-flick method.*

**Reviewer #2:**

Suggested minor corrections have been indicated on the text, these corrections should be taken into consideration.

*We have reviewed the corrections indicated in the text, thank you for your suggestions*

Conclusion section of the manuscript may be extended. Some suggestion for future may be added to the conclusion section of the manuscript

*Thank you for the input, we have added suggestion regarding future studies in line 283–9.*

*We would also like to address the concern of reviewer 2’s answer regarding the editorial question of ‘Have the authors made all data underlying the findings in their manuscript fully available?’ Which data underlying the findings weren’t available according to reviewer 2 so that we could improve?*

**Reviewer #3**

As you mentioned in your article sample size of the study is very small. But if your study made larger, its validity may become more appropriate. But you also mentioned the small sample size in your study. I recommend planning a study with a sufficiently large sample size.

*We agree and aware of the issue regarding the small sample size in this current small study, but for a pilot study and technique demonstration purpose, we hope that this study could show the feasibility of the proposed technique (Line 272–3). In the near future, we planned to publish our study which utilize this technique, with larger sample size and thus better validity, with a main purpose of therapeutic intrathecal injection in neuropathic rats model.*

We thank the reviewers for their time and input regarding this manuscript. Please address further queries concerning this manuscript to Pryambodho at pry@cbn.net.id. We look forward to hear from you at your earliest convenience. Thank you for your consideration and attention on this manuscript.

Regards,

Pryambodho, MD

Department of Anesthesiology and Intensive Care,

Faculty of Medicine, Universitas Indonesia,

Jakarta, Indonesia
